# Incorporating Data From Text Messages and Email Into Psychotherapy: Randomized Controlled Trial

**DOI:** 10.2196/93446

**Published:** 2026-10-19

**Authors:** Ipsit V Vahia, Julia Kimball, Boyu Ren, Hailey Cray, Rebecca Dickinson, Heejung Kim, Dylan X Guan, Kerry J Ressler

**Affiliations:** 1 McLean Hospital, 115 Mill Street, Belmont, MA, 02478, United States, 1 617 855 3267; 2 Department of Psychiatry, Harvard Medical School, Boston, MA, United States; 3 VA Boston Healthcare System, Boston, MA, United States; 4 School of Nursing, Yale University, New Haven, CT, United States; 5 School of Medicine, Nova Southeastern University, Davie, FL, United States; 6 School of Medicine, University of Calgary, Calgary, AB, Canada

**Keywords:** psychotherapy, text messaging, collateral information, electronic media, depression, anxiety

## Abstract

**Background:**

As digital communication becomes central to daily life, psychotherapy increasingly has access to patients’ electronic media data. Although digital phenotyping has been widely studied, less is known about whether incorporating personal communication data, such as text messages, improves clinical outcomes in psychotherapy.

**Objective:**

This study aimed to determine whether integrating personalized text message data into psychotherapy improves depression, anxiety, health-related quality of life, and therapeutic alliance and to examine whether increased access to collateral information influences clinical decision-making.

**Methods:**

The Electronic Media and its Impact on Psychotherapy (EMAP) study was a randomized controlled trial conducted in adult and geriatric outpatient and partial hospitalization settings at an academic psychiatric hospital. Participants receiving psychotherapy for primary depression or anxiety (n=101) were randomized to electronic media–enhanced therapy (EME; n=52) or treatment as usual (TAU; n=49). In EME, research staff reviewed participants’ text messages prior to sessions using personalized mood-related key terms and presented aggregated findings on a Health Insurance Portability and Accountability Act (HIPAA)–compliant clinician dashboard. TAU participants received standard care without message review. Outcomes included change scores for the Patient Health Questionnaire-9 (PHQ-9), the Generalized Anxiety Disorder-7 (GAD-7), the 36-item Short Form Health Survey (SF-36), and the Working Alliance Inventory–Short Revised (WAI-SR). Linear regression and nonparametric tests compared groups. Moderation analyses assessed whether the frequency of information access influenced treatment changes.

**Results:**

No statistically significant between-group differences were observed in anxiety or working alliance. Among participants with SF-36 follow-up data (n=65), EME participants demonstrated greater improvement in the pain subscale. Greater frequency of information access was associated with increased treatment-related changes in the EME group. Access to additional collateral information was associated with more clinical actions but not with improved distal outcomes.

**Conclusions:**

Integrating personalized text message data into short-term psychotherapy did not significantly improve depression or anxiety, and it neither improved nor worsened therapeutic alliance. However, access to greater amounts of collateral information, including electronic media, influenced clinical decision-making, suggesting nuanced and context-dependent effects.

## Introduction

Over the past decade, the digitization of everyday life has meant that devices such as smartphones serve as conduits for everyday function and communication [1]. Interpersonal communication now includes digital media such as text messages, blogs, emails, and social media; much of this communication is chronicled and preserved in ways that did not exist until the advent of these technologies. Increasingly, psychiatry, and particularly the realm of psychotherapy, has begun to recognize the ways in which digital data can be incorporated into care. A vast literature exists in the domain of digital biomarkers and digital phenotyping [2-5], where sensors and devices track behavior and, in turn, generate objective measures of activity, sleep, mood, and parameters such as medication responses and side effects. However, a simpler and more intuitive use of this digital collateral information available through digital media and communication—such as text messages—has been relatively understudied.

Psychotherapy relies on patient recall of key information, events, and interactions. There is evidence to indicate that clinicians sporadically incorporate information from digital communications into therapy sessions and that including this information may have a positive impact on the psychotherapeutic process [6-8]. However, emerging evidence also indicates that the effectiveness of these approaches may be situational and that outcomes may vary depending on how data are presented and accessed. For instance, one study [9] found that the use of a dashboard that presented clinicians with broad metrics, such as time spent on a smartphone during the night, miles walked per week, and top searches on social media, led to no statistically significant changes in patients’ well-being scores. A follow-up study [10] also found that clinician use of such a digital dashboard did not improve well-being. Notably, both studies found that patients and clinicians alike appreciated the awareness the dashboard brought to patients’ digital use. Patients also reported feeling comfortable and positive about the dashboard experience [10]. This suggests that the impact of this approach may be subtle and nuanced, a notion that is being increasingly replicated in other studies [11]. However, neither study investigated whether there was a differential impact from evaluating more personal communication, such as email or text messages. Investigating this is relevant because Liu et al [12] demonstrated that the language in text messages strongly correlates with the severity of self-reported depressive symptoms. This finding was based on sentiment analysis of existing text messages; however, how it may translate into real-time inclusion of text messaging content into psychotherapy is unknown.

There are important implications for gaining a more sophisticated understanding of how access to broader information from digital communication and media impacts outcomes in psychotherapy. Spending time in a session reviewing digital content can take away from time spent engaged directly with patients [13], and it is not clear how this may impact rapport. It is also not clear whether any benefits are empirical or situational. Finally, because there is great interindividual variance in how individuals across the age span express emotion in electronic communication [14], there is a need for guidelines around how to optimize the use of electronic media. To address these knowledge gaps, we conducted a randomized controlled trial of an intervention in which research staff performed a personalized review of patients’ text message communication prior to a psychotherapy session. This information was aggregated into an interactive dashboard for clinicians to access during their sessions with patients. We sought to assess whether (1) access to collateral information about text communication impacted depression and anxiety, (2) using a dashboard to review this information during therapy sessions impacted the therapeutic alliance, and (3) accessing more collateral information, including digital communication, during therapy sessions impacted the way therapists planned treatment for each session.

We hypothesized that patients receiving care incorporating electronic media would experience greater reductions in depression and anxiety, improved health-related quality of life scores, and a stronger working alliance compared with those receiving treatment as usual (TAU). Additionally, we explored how clinicians used data during sessions and whether increased data access influenced their approach to care.

## Methods

### Participants

This study was conducted in the adult and geriatric outpatient and partial hospitalization programs at McLean Hospital, an academic psychiatric hospital affiliated with Mass General Brigham Health and Harvard Medical School. Recruitment occurred from July 2020 to July 2023. We initially contacted clinicians providing outpatient psychotherapy, including psychiatrists, psychologists, and social workers. Clinicians were eligible if they offered psychotherapy, regardless of whether they also prescribed medications concomitantly. On the basis of referrals from clinicians, we recruited individuals engaged in individual psychotherapy for primary diagnoses of anxiety or depression. Individuals with cognitive impairment, active psychosis, or active substance use disorders were excluded, since this study focused primarily on psychotherapy outcomes for depression and anxiety. Of note, the study protocol was originally designed prior to the COVID-19 pandemic but was adapted since the pandemic-related lockdown was imposed shortly after the study was approved by the institutional review board (IRB). Protocols were adjusted to allow for recruitment and administration of the study procedures virtually, whereas the original protocol was designed for in-person visits. Study visits occurred between August 2020 and July 2023.

### Intervention

Participants in this study were randomized into 2 groups using randomized number sequencing: the electronic media–enhanced therapy (EME) group and the TAU group. Participants were assigned in a 1:1 ratio between groups using a pregenerated random allocation sequence. Participants were assigned sequentially according to this randomization list as they were enrolled. The study was not blinded, and allocation was not concealed. The EME group received therapy augmented by the collection and analysis of electronic media data, while the TAU group received standard clinical treatment without the incorporation of electronic media data. Both groups were administered standardized scales. For both the EME and TAU groups, research assistants met with participants prior to each scheduled clinical session. During these meetings, which lasted approximately 30 minutes, study staff administered questionnaires assessing participants’ use of messaging apps (eg, text messages, Facebook Messenger, and WhatsApp). The questionnaires included both objective measures (eg, frequency and number of active conversations) and subjective items assessing participants’ comfort with the research process.

In the EME group, research assistants also reviewed participants’ messaging content using Zoom’s screen-sharing feature. Participants were instructed on how to share their screens and were asked to search their messages for 12 preselected key terms that reflected emotional valence for mood states such as depression and anxiety. These terms were chosen based on consensus of the research team, informed by both clinical experience and prior research. In consultation with the participant’s clinician, each participant was provided with a personalized list of 12 key terms, which were updated as necessary. For participants in partial hospitalization programs, key terms were identified within the previous day’s messaging to account for the shorter duration between clinical visits. The standardized scales administered to both groups were as follows: (1) the Patient Health Questionnaire-9 (PHQ-9) [15], (2) the Generalized Anxiety Disorder-7 (GAD-7) [16], (3) the Working Alliance Inventory–Short Revised (WAI-SR) [17], (4) the RAND Medical Outcomes Study 36-item Short Form Health Survey (SF-36) [18], and (5) the McLean Collateral Information and Clinical Actionability Scale (M-CICAS) [7]. Collected data were entered into a Health Insurance Portability and Accountability Act (HIPAA)–compliant platform developed by Mirah, a Boston-based company. Mirah is an established digital behavioral health platform that provides software for the secure delivery of measurement-based care tools and patient-reported outcomes. For this study, Mirah custom-designed a portal that enabled data collection, data storage, and a clinician-facing dashboard that presented aggregated data from messages as well as the standardized scales. Clinicians reviewed the data before or during psychotherapy sessions. Any content that raised clinical concerns during presession data collection was forwarded to the principal investigator (PI) and the participant’s therapist for further evaluation and action. Since all psychotherapy sessions were conducted in person or virtually at McLean Hospital, a well-established infrastructure and protocols were in place to manage any decompensation or severe symptoms and would be activated at the clinicians’ discretion.

In the TAU group, no review of messaging content occurred. Instead, research assistants focused on collecting self-reported data on mood and health functioning, which were also tracked using the same Mirah platform.

### Research Assistant Training

Before engaging in data collection, all research assistants underwent training to ensure interrater reliability. The training, conducted by the PI or a clinical coinvestigator, involved a certification process requiring research assistants to identify 10 emotionally charged sentences out of 20 conversational samples with at least 90% accuracy. Research assistants were also trained to detect content that might raise safety concerns and to follow appropriate procedures for managing these concerns. Once certified, research assistants were authorized to record relevant data, including the frequency of key terms used in participants’ messaging, the emotional tone of these messages, and the identities of the individuals involved in the conversations.

### Assessments

During therapy sessions, clinicians in both groups reviewed the mood scales, health data, and collected collateral information, including messaging behavior for participants in the EME group. Clinicians were encouraged to incorporate this information into their clinical decision-making but retained discretion over how to use the data. In the EME group, clinicians could choose to view specific messaging content, especially if there were concerns about suicidality, mood changes, or disorganized thoughts. Any emergent concerns related to safety or mental health were immediately triaged by the therapist or a licensed clinician on the study team, with the PI available for further consultation if necessary.

After each clinical session, both patients and clinicians completed assessments. The WAI-SR was administered to clinicians and patients at baseline and final visits to assess the therapeutic alliance.

For participants in partial hospitalization programs, the assessment schedule was adjusted to account for their shorter treatment duration. In these cases, mood and health questionnaires were administered between daily clinical sessions.

### Ethical Considerations

The Electronic Media and its Impact on Psychotherapy (EMAP) study was registered with ClinicalTrials.gov (NCT03712267) and approved by the Mass General Brigham Institutional Review Board (2018P002179). All participants provided informed consent using a standard IRB-approved form and protocol. Participants were compensated US $25 per study visit for their time spent with research assistants reviewing messaging content or completing questionnaires. Study clinicians were compensated US $15 per session for the additional time required to complete the study-related questionnaires.

All collected data, including messaging content and mood assessments, were securely stored on the Mirah platform. Participants retained full autonomy over access to their devices. Research staff did not have access to participants’ passwords or other security credentials, and participants determined which messages were shared. Any decision to withhold specific messages was respected and formally documented.

When study staff or clinicians identified content suggesting potential safety concerns (eg, indications of suicidality, psychosis, or marked mood changes), these concerns were promptly escalated to the participant’s therapist and the study PI for immediate evaluation and intervention. The study complied with all applicable mandated reporting requirements, and any adverse events were reported to the IRB.

### Statistical Analysis

Demographic characteristics were summarized using means, SD, and ranges for continuous variables and frequencies and proportions for categorical variables. Treatment effects were evaluated using change scores for each outcome (SF-36, WAI-SR, PHQ-9, and GAD-7), calculated as the difference between scores at the last session and the first session. SF-36 subscale change scores were modeled as a function of treatment group (EME intervention vs TAU control) using linear regression, controlling for the total number of sessions. This approach was repeated for the PHQ-9 and GAD-7 outcome measurements. Because WAI-SR change scores violated normality assumptions, Wilcoxon rank-sum tests were used for this outcome. Secondary analyses were conducted within the subsample of participants enrolled in the partial hospitalization program. Finally, moderation analyses were performed to determine whether treatment group (EME vs TAU) modified the association between frequency of information access and treatment changes using the M-CICAS measures. Given the small sample size, no adjustments for multiple comparisons were applied. Statistical significance was defined as *P*<.05 for all tests. All analyses were conducted using SPSS (version 26.0; IBM Corp).

## Results

The study included 101 participants, with 52 (51.5%) assigned to the EME intervention group and 49 (48.5%) to the TAU control group (Figure 1). Most participants (n=87, 86.1%) were enrolled in a partial hospitalization program. Across the cohort, the mean age was 39.4 (SD 16.2) years, 63.4% (n=64) identified as female, and 80.2% (n=81) self-identified as White (Table 1). Participants reported an average of 16.3 (SD 2.3) years of education. The most common primary psychiatric diagnoses were depression (n=75, 74.3%) and anxiety (n=22, 21.8%). No significant differences were observed between the EME and TAU groups across demographic variables. The mean PHQ-9 score in our sample was 10.85 (SD 5.49; range 1‐23; median 10.5, IQR 8-16) and the mean GAD-7 score was 8.43 (SD 4.62; range 0‐20; median 8, IQR 5-12), indicating that the overall sample demonstrated at least moderate depression and anxiety severity. There were no significant differences between the EME and TAU groups in the severity of anxiety and depression. Because all participants were recruited with clinician input during the course of ongoing psychotherapy or as part of a partial hospital program, we did not lose any participants to attrition, and data from all participants were included in analyses.

**Figure 1.**
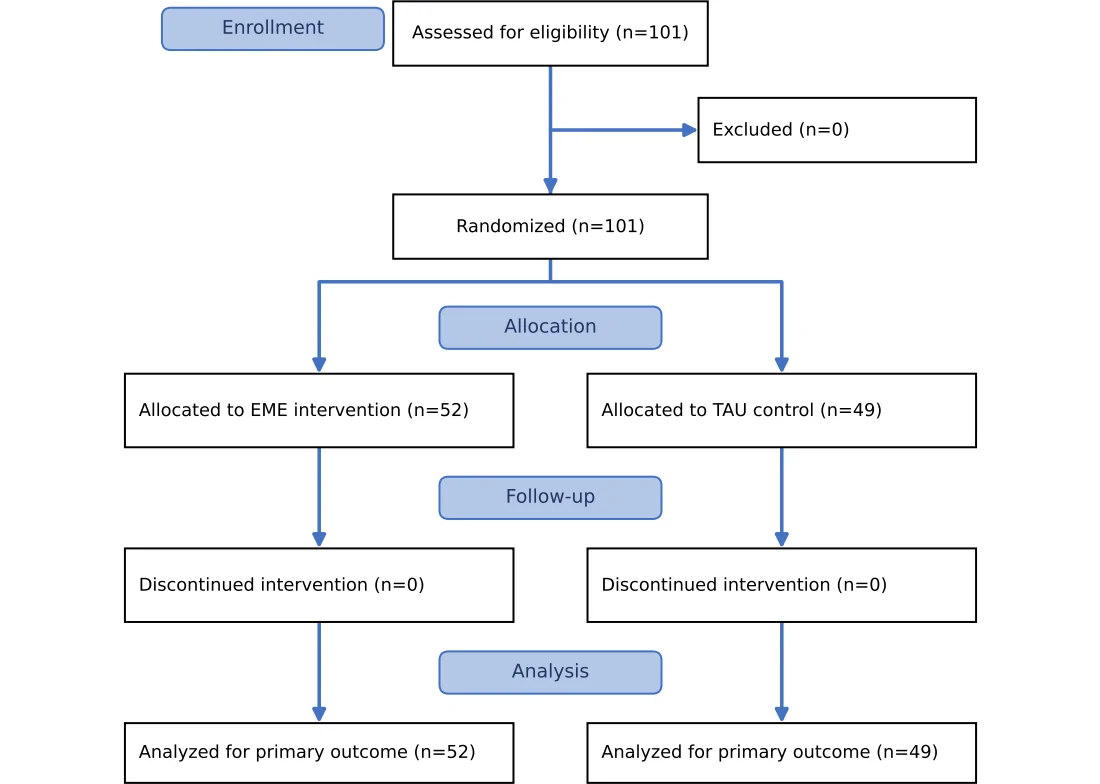
CONSORT (Consolidated Standards of Reporting Trials) flow diagram. Participants were only assessed for eligibility based on referral from their treating clinicians during the course of ongoing psychotherapy or as part of a partial hospital program. Referring clinicians were familiar with the study protocol and only referred patients whom they deemed as good fits for the study. This process resulted in a sample that was clinically prescreened, and their ongoing engagement in clinical care meant that we did not lose any participants to attrition, and data from all participants were included in analyses. EME: electronic media–enhanced therapy; TAU: treatment as usual.

**Table 1.** Sample characteristics at baseline.

Variables	Total	EME^a^	TAU^b^
Age (y), mean (SD; range)	39.4 (16.2; 19‐80)	40.6 (16.3; 19‐70)	38.2 (16.2; 19‐80)
Sex, n (%)			
Female	64 (63.4)	30 (57.7)	34 (69.4)
Male	25 (24.8)	17 (32.7)	8 (16.3)
Other	4 (4)	2 (3.8)	2 (4.1)
Missing	8 (7.9)	3 (5.8)	5 (10.2)
Education (y), mean (SD; range)	16.3 (2.3; 12‐22)	16.7 (2.4; 12‐22)	15.9 (2.0; 12‐20)
Race, n (%)			
White	81 (80.2)	43 (82.7)	38 (77.6)
Black or African American	3 (3)	0 (0)	3 (6.1)
Asian	5 (5)	3 (5.8)	2 (4.1)
Other	8 (7.9)	6 (11.5)	2 (4.1)
Missing	4 (4)	0 (0)	4 (8.2)
Diagnosis, n (%)			
Depression	75 (74.3)	38 (73.1)	37 (75.5)
Anxiety	22 (21.8)	13 (25)	9 (18.4)
Missing	4 (4)	1 (1.9)	3 (6.1)

a EME: electronic media–enhanced therapy.

b TAU: treatment as usual.

Participants in the EME group exhibited greater reductions in PHQ-9 and GAD-7 scores from their first to last session compared with those in the TAU group; however, these differences were not statistically significant (PHQ-9 b=–0.62, 95% CI –2.87 to 1.62; *P*=.58; GAD-7 b=–0.26, 95% CI –1.90 to 1.39; *P*=.75). Among the subset of 65 participants with follow-up SF-36 data, no significant differences were observed between groups across SF-36 domains, except for pain (Table 2): EME participants demonstrated a 9.54-point greater increase in SF-36 pain subscale scores compared with TAU participants (95% CI 1.94-17.14; *P*=.02). This effect persisted within the subsample enrolled in the partial hospitalization program (b=8.55, 95% CI 0.55-16.55; *P*=.04).

Wilcoxon rank-sum tests further revealed no group differences in changes in WAI-SR item scores (Table 3) among participants with available follow-up data (n=41).

**Table 2.** Change in SF-36 subscales between first and last session across electronic media–enhanced therapy and treatment as usual groups.

SF-36^a^ subscale	b (95% CI)^b^	*P* value
Full sample^c^		
Emotional well-being	–2.09 (–8.60 to 4.42)	.53
Energy or fatigue	3.77 (–3.19 to 10.73)	.29
General health	0.27 (–5.45 to 5.99)	.93
Pain	9.54 (1.94 to 17.14)	.02
Physical functioning	0.70 (–4.65 to 6.05)	.80
Role limitations		
Emotional	0.71 (–9.52 to 10.94)	.89
Physical	1.50 (–12.49 to 15.49)	.83
Social functioning	2.42 (–7.01 to 11.85)	.62
Partial program^d^		
Emotional well-being	–3.27 (–10.13 to 3.59)	.35
Energy or fatigue	5.18 (–2.09 to 12.45)	.17
General health	0.10 (–5.56 to 5.76)	.97
Pain	8.55 (0.55 to 16.55)	.04
Physical functioning	2.11 (–3.40 to 7.62)	.46
Role limitations		
Emotional	1.76 (–9.29 to 12.81)	.76
Physical	3.98 (–10.66 to 18.62)	.60
Social functioning	1.40 (–8.60 to 11.40)	.79

a SF-36: 36-item Short Form Health Survey.

b b coefficients were estimated from linear regression. Models were adjusted for the number of total sessions received.

c Participants with follow-up SF-36 data (n=65).

d Subgroup of participants in the partial hospitalization program with follow-up SF-36 data (n=60).

**Table 3.** Change in Working Alliance Inventory–Short Revised (WAI-SR) item scores between first and last session across electronic media–enhanced therapy and treatment as usual groups.

WAI-SR item	Mean difference	*P* value
Full sample^a^		
As a result of these sessions I am clearer as to how I might be able to change	–0.39	.26
What I am doing in therapy gives me new ways of looking at my problem	–0.13	.61
I believe (therapist) likes me	0.24	.10
(Therapist) and I collaborate on setting goals for my therapy	0.03	.47
(Therapist) and I respect each other	0.04	.45
(Therapist) and I are working towards mutually agreed upon goals	0.10	.74
I feel that (therapist) appreciates me	–0.26	.38
(Therapist) and I agree on what is important for me to work on	0.09	.81
I feel (therapist) cares about me even when I do things that he/she does not approve of	–0.04	.91
I feel that the things I do in therapy will help me to accomplish the changes that I want	0.32	.26
(Therapist) and I have established a good understanding of the kind of changes that would be good for me	–0.29	.17
I believe the way we are working with my problem is correct	0.10	.99
Partial program^b^		
As a result of these sessions I am clearer as to how I might be able to change	–0.43	.21
What I am doing in therapy gives me new ways of looking at my problem	–0.09	.75
I believe (therapist) likes me	0.25	.10
(Therapist) and I collaborate on setting goals for my therapy	0.10	.30
(Therapist) and I respect each other	0.04	.45
(Therapist) and I are working towards mutually agreed upon goals	0.26	.40
I feel that (therapist) appreciates me	–0.23	.50
(Therapist) and I agree on what is important for me to work on	0.14	.63
I feel (therapist) cares about me even when I do things that he/she does not approve of	–0.09	.77
I feel that the things I do in therapy will help me to accomplish the changes that I want	0.43	.15
(Therapist) and I have established a good understanding of the kind of changes that would be good for me	–0.22	.25
I believe the way we are working with my problem is correct	0.16	.82

a Participants with follow-up WAI-SR data (n=41).

b Subgroup of participants in the partial hospitalization program with follow-up WAI-SR data (n=39).

The association between treatment changes and the frequency of information access differed by assignment to the EME or TAU arms (*P*=.02). Specifically, treatment changes were positively associated with the number of times information was accessed in the EME group (b=0.72), but not in the TAU group (b=–0.01, 95% CI 0.12-1.34; *P*=.02; Figure 2). We also noted that no participants expressed reservations about allowing clinicians to read their actual text messages when requested.

**Figure 2.**
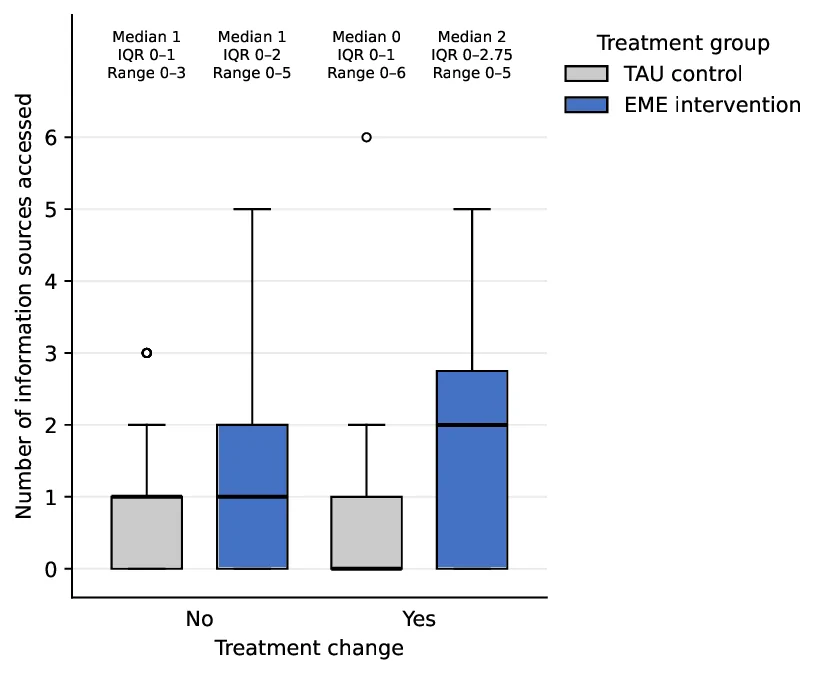
Distribution of the number of sources of information accessed by treatment group and treatment change status. EME: electronic media–enhanced therapy; TAU: treatment as usual.

## Discussion

This study examined whether incorporating data from electronic media into psychotherapy influences clinical outcomes and the therapeutic alliance between clinicians and patients. It also explored how integrating data from multiple sources informs clinical decision-making. At a time when ever-growing streams of data are becoming accessible to clinicians, this study addresses the fundamental question of whether and how these data can add value. In this study of 101 clinician-patient dyads, no statistically significant differences were observed in PHQ-9 or GAD-7 scores between the EME and TAU groups, suggesting that incorporating electronic media did not improve distal clinical outcomes related to anxiety and depression. Similarly, WAI-SR item scores did not differ between groups, indicating that the therapeutic alliance was unaffected by the inclusion of electronic media. Consequently, the study’s primary hypotheses were not supported. We did note a significant improvement in SF-36 pain subscale scores. This finding was unexpected and was not hypothesized, since the primary intervention did not target pain. This finding merits replication in other clinical populations, since the CI is wide.

Exploratory analyses indicated that accessing a greater number of information sources was associated with an increased number of clinical actions taken during visits. This finding encompassed all collateral information sources, not solely digital data. However, taking more clinical actions did not translate into improved outcomes when comparing groups. Nonetheless, this exploratory finding suggests that clinicians tend to make more changes during psychotherapy when provided with additional collateral information. One possible reason for the lack of support for the primary hypothesis may relate to characteristics of the study sample, as most participants were enrolled in a partial hospitalization program and received care for only 2 weeks, during which we gathered data for an average of fewer than 4 sessions per participant. This may simply not have been sufficient contact to capture change in downstream variables. However, at the individual level, there may be benefits to more clinical actions from accessing more data that are not captured by our selected outcome variables. These findings are consistent with existing studies [9,10] that provided clinicians with access to a dashboard displaying data from patients’ social media and internet use. These studies found that while providing clinicians with this dashboard was feasible and responses to dashboard access were generally positive, distal clinical outcomes did not improve. Our finding also provides a counterpoint to an extant literature that has suggested that access to informatics may universally improve outcomes [19].

This study has several limitations that must be factored into interpretation. We aimed to make this study as aligned with the broadest possible range of psychotherapy approaches as possible. In doing so, we limited the standardization of the treatment protocols. This heterogeneity may have influenced the lack of impact on the primary outcomes. Also, in personalizing search terms for each participant, we aimed to capture the fact that the expression of emotion may be different for different individuals—but this meant that the search terms were not standardized. Moreover, in giving clinicians broad leeway in how to use the data from text messaging, our design introduced variability that we were not able to fully adjust for in the analysis. Additional variability may have been introduced by the fact that the number of sessions per participant ranged from 4 to 13.

This project highlights several meaningful directions for future research. Chief among these is that there may be subtleties in the ways that accessing a person’s electronic media can impact psychotherapy, and these subtleties are likely specific to each individual. Although the primary hypotheses were not supported, the exploratory findings suggest that providing clinicians with additional collateral information, including digital data, can influence clinical decision-making [20]. However, broad outcomes such as improvement in depression or anxiety, particularly in a study where participants were tracked for as few as 2 weeks, may not capture this impact. We recommend that future work incorporate novel designs that can capture intraindividual changes in response to access to electronic media for psychotherapy.

## Supplementary material

10.2196/93446 Checklist 1 CONSORT-eHEALTH checklist (V 1.6.1).

## Abbreviations

EMAP: Electronic Media and its Impact on Psychotherapy
EME: electronic media–enhanced therapy
GAD-7: Generalized Anxiety Disorder-7
HIPAA: Health Insurance Portability and Accountability Act
IRB: institutional review board
M-CICAS: McLean Collateral Information and Clinical Actionability Scale
PHQ-9: Patient Health Questionnaire-9
PI: principal investigator
SF-36: 36-item Short Form Health Survey
TAU: treatment as usual
WAI-SR: Working Alliance Inventory–Short Revised
